# 3PD: Rapid design of optimal primers for chromosome conformation capture assays

**DOI:** 10.1186/1471-2164-10-635

**Published:** 2009-12-29

**Authors:** Sebastian Fröhler, Christoph Dieterich

**Affiliations:** 1 Berlin Institute for Medical Systems Biology at the Max Delbrück Center for Molecular Medicine, Robert-Rössle-Strasse 10, 13125 Berlin-Buch, Germany

## Abstract

**Background:**

Higher eukaryotes control the expression of their genes by mechanisms that we are just beginning to understand. A complex layer of control is the dynamic spatial organization of the nucleus.

**Results:**

We present a bioinformatics solution (3PD) to support the experimentalist in detecting long-ranging *intra *or *inter *chromosomal contacts by Chromosome conformation capture (3C) assays. 3C assays take a snapshot of chromosomal contacts by a fixation step and quantify them by PCR. Our contribution is to rapidly design an optimal primer set for the crucial PCR step. Our primer design reduces the level of experimental error as primers are highly similar in terms of physical properties and amplicon length. All 3C primers are compatible with multiplex PCR reactions. Primer uniqueness is checked genome-wide with a suitable index structure.

**Conclusions:**

In summary, our software 3PD facilitates genome-wide primer design for 3C experiments in a matter of seconds. Our software is available as a web server at: http://www.pristionchus.org/3CPrimerDesign/.

## Background

Gene transcription is regulated by several means involving different levels of complexity in multicellular eukaryote genomes. Most attention has been paid to local protein/DNA interaction ever since Britten and Davidson had published their ground-breaking paper on gene regulation [1]. Regulatory RNA, chromatin-and histone-modifying complexes have recently gained much attention [2]. Remarkably, we are just beginning to understand the impact of genome organization in space and time on gene regulation [3]. Several reports have been published that underline the importance of dynamic spatial organization of the nucleus in development and differentiation [3]. Chromosome conformation capture assays help to decipher long-range *intra*- or *inter*-chromosomal interactions and deliver an image of genome plasticity. Up to date, no bioinformatics solution has been provided to support experimentalists in the layout of 3C experiments [4,5]. It has been pointed out before that the design of equally efficient primers is fundamentally important to the success of 3C experiments [6]. We present a computational resource for rapid 3C primer design. Our approach takes several design constraints into account that are either common to primer design in general (uniqueness in the genome, low self-complementarity and similar physical properties like melting temperature) or specific to the 3C method in particular (amplicon length, spacing and 'unsafe' misprimings). Our web application chooses the best primer sequences for any given genomic region of interest and any suitable restriction enzyme. Our software seeks to maximize a score function, which encompasses all aforementioned design constraints.

We will now briefly review the relevant experimental technique and continue with an overview of our approach.

### Chromatin Conformation Capture (3C)

**Figure 1:**
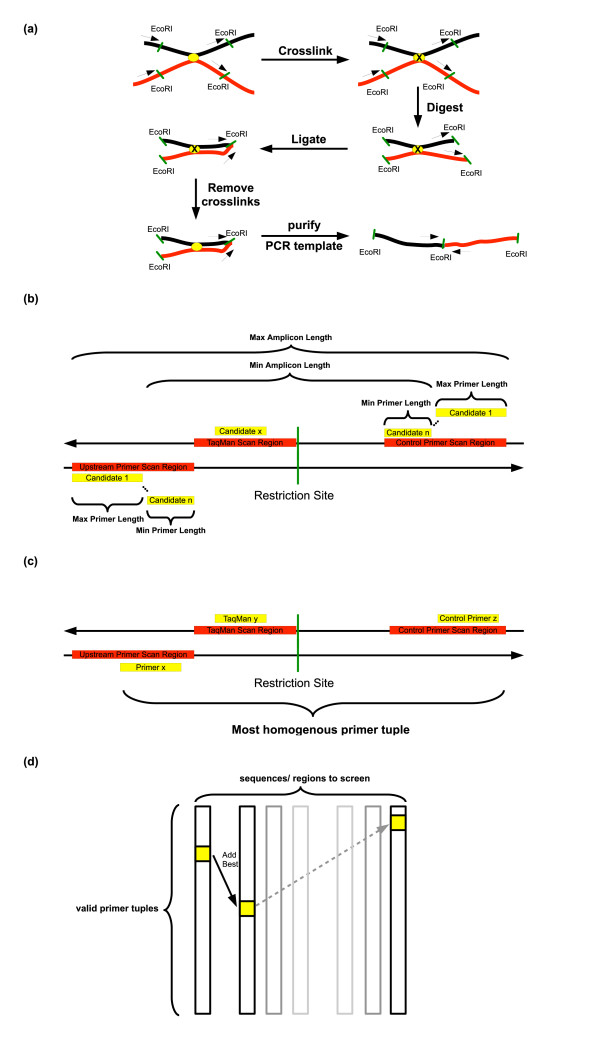
**Primer Design for 3C Experiments**. (a): The important steps of Chromosome Conformation Capture (3C): (1) Interacting DNA segments (red and black) are crosslinked. (2) Crosslinked DNA segments are cut with a restriction enzyme (EcoRI in this example, restriction sites shown as green lines). (3) Segments are religated at low DNA concentration strongly favoring intramolecular ligations between crosslinked segments compared to other, free floating segments. (4) Crosslinks are removed. (5) Hybrid templates are purified and quantified by quantitative PCR. After digestion (step 2), this figure suggests the possibility of four unique ligation products of the four DNA ends - each resulting in a ligation product between the red and the black fragment. Since only upstream primers are designed and used for the 3C method itself, out of these four products, only one is amplified. Positions of possible upstream primers are denoted by black arrows. Downstream primers, as designed by our program should be used to ckeck proper digestion of each site to be studied! **(**b): Graphical representation of some primer search parameters. Minimum and maximum amplicon lengths are shown depending on minimum and maximum primer lengths. All parameters are specified by the user. **(**c): From the list of candidate primers as obtained in (b), for each scan region, primer tuples are created, checked and scored. This figure shows the optimal primer pair for the current restriction site. Primers and primer tuples are scored by the scoring functions depicted in Figure 3. (d): In order to retrieve the optimal set of primer tuples, one for each sequence region to be scanned, the lists of acceptable primer tuples (shown as columns) are screened. A candidate primer tuple set corresponds to a path through the matrix of candidate tuples from left to right. It should be noted that this tuple set contains exactly one primer tuple for each scan region to be screened. In each step, the primer tuple which is most homogeneous to the current set is added. At the end, the tuple set is scored and the optimal scoring tuple set is returned.

The 3C methodology was developed by Dekker *et al. *[4] and allows for the specific determination of DNA/DNA contacts, which are possibly separated by a long distance on the primary sequence. These contacts may be transient and are fixed by a crosslinking reaction using formaldehyde. The cross-linked DNA is digested with a restriction enzyme of class II, which produces symmetric sticky ends. These cross-linked restriction fragments are ligated at low DNA concentrations. Finally, crosslinking is reversed by digesting proteins with Proteinase K and phenol/chloroform extraction. Selected ligation products (chimeric DNA template) are quantified by a subsequent quantitative PCR step. Only one out of four possible ligation products is amplified since all are assumed to be equally frequent (see also Figure 1a). It should be noted that this assumption only holds if the crosslink is in the center of both dna fragments - an information typically not available a-priori! A 'negative' control reaction is effected by omitting the crosslinking step. Significant differences in crosslinking frequencies between sample and control template are candidate regulatory DNA contacts [4]. Ultimately, a three dimensional (3D) model of the genomic region of interest may be built by translating crosslinking frequencies into physical distances [4]. All details about the 3C protocol can be found in [5].

### Requirements for 3C Primer Design

Several requirements have to be met when designing primers for the 3C method. First, standard requirements for PCR primer design need to be satisfied. A commonly accepted set of standard requirements for PCR primer design is implemented in the 'primer3' software [7], which is frequently used for singleplex primer pair design. Second, some additional constraints, which originate from the 3C methodology, have to be fulfilled.

For standard PCR, primers have to be unique to avoid mispriming events. Consequently, each primer hybridizes to only one specific location on the DNA sequence of interest. Additionally, primers should not be self-complementary, which would result in primer dimers or hairpin structures. In case of designing more than one primer, each primer must not hybridize to any other primer in the same reaction. In other words, primers should be compatible with multiplex-PCR reactions. A violation of these requirements can lead to poor PCR efficiency and, in the worst case, can result in false-positive amplicons. For example, a false-positive amplicon can occur if the 3'-end of one primer is self-complementary. The primer will bind to itself and will produce very short PCR products. Another aspect concerns the physical properties of primers namely: GC-content and melting temperature. For maximum PCR efficiency, all primer pairs should have similar physical properties. Typically, a primer set is considered to be acceptable if the GC-content ranges between 40 and 60 percent and if the melting temperatures does not differ by more than five degrees Celsius between primers. Ideally, for quantitative PCR, primer melting temperatures should not differ by more than two to three degrees Celsius.

Besides these general requirements, there are some additional constraints for primer design imposed by the 3C method (Figure 1b). Firstly, all primers should have an equal distance to their closest restriction site. This is important to avoid any amplification bias, which may arise from different amplicon lengths. A suitable amplicon length for quantitative PCR is ~100 basepairs. For high amplification efficiencies, amplicon lengths should not exceed 200 basepairs [8]. The choice of restriction enzyme should be flexible in order to pick the most suitable enzyme in a sequence- and task-specific manner. Secondly, no mispriming events should take place close to any restriction site anywhere in the genome. This could lead to false positive amplicons after the re-ligation step and - in the worst case - could completely mask the signal. Below a certain length, those amplicons are hard to discern from true positive ones. Finally, since 3C determines interaction frequencies of DNA segments, it is important that primers (and their restriction sites) are equally distributed across the genomic region of interest. Ideally, the distance between two adjacent primers should be equal for all selected primers.

In standard primer design, PCR primers are first designed and later checked for mispriming. Many experimentalist do this manually - if at all. Our primer design approach is fully automated, integrates mispriming checks and produces efficient primer sets, without any need for human intervention. It should be noted that an extended mispriming check is required for the 3C method since hybrid templates are quantified. This leads to a combinatorial explosion of potential misprimings which can impair quantification of the 'real' priming.

The 3C procedure is continuously improved by researchers world-wide. In early 3C experiments, ligation products were quantified by gel image analysis, which is error-prone. Dekker [5] and others improved on that by using quantitative TaqMan PCR to quantify ligation products. They also suggested to use control primers to assess the digestion efficiency for any particular locus. However, TaqMan probes are costly. This is not a problem for one-to-many experiments where one locus is tested against many other loci, but it is for rigorous many-to-many experiments where all pairwise interactions are analyzed. Hassan *et al. *[9] quantify ligation products with a SYBR-Green-based method, which eliminates the need of TaqMan probes. They invented a technique called quantitative melting curve analysis, which is not hampered by the high fluorescence background of a standard *C*_*t *_analysis. The method we introduce here is able to create primer pairs suitable for all three variants of 3C: the classic 3C method quantified on gel, the 3C variant using TaqMan probes for quantification and the newly invented 3C protocol using melting curve quantification. In the following, we will give a detailed description of the components of our method in the 'Implementation' section. We will then evaluate the performance of our method on a real-world example in the 'Results and Discussion' section.

## Implementation

We designed a computer program for 3C PCR primer design that fulfills all aforementioned constraints. Our program can be used in two different modes: global mode, most useful for unbiased 3D structure modeling, and targeted mode, to be used for targeted detection of candidate interactors. Depending on the design setup specified by the user, some steps of the algorithm are omitted (like the TaqMan probe design, in case only upstream and downstream primers are required by the user, or the selection of homogeneously distributed restriction sites, in case a targeted screen for candidate interactors is performed). The program flow can be separated into three key steps:

1. Selection of homogeneously distributed restriction sites for the restriction enzyme specified by the user (in 'global mode' only)

2. Enumeration and validation of candidate primers in the vicinity of these restriction sites (depending on design task, two or three primers are designed around each restriction site)

3. Picking of the most-homogeneous set of primer tuples, one tuple for each restriction site.

**Figure 2:**
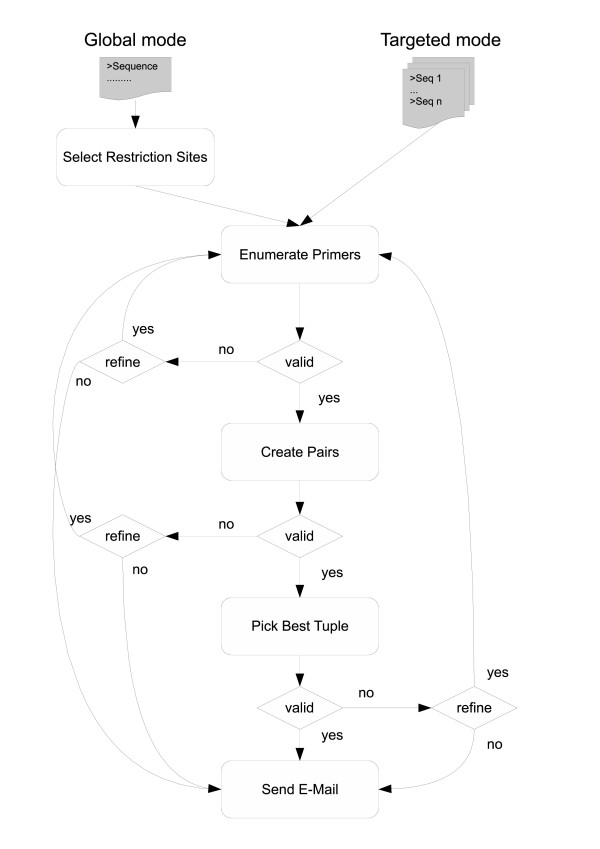
**Primer Design Flowchart**. A flowchart visualizing the main steps in our program. Starting with one sequence ('global mode') or several sequences ('targeted mode'), primers are enumerated and pairs are created. Thereafter, the most-homogeneous tuple set from the lists of tuples is determined containing exactly one tuple from each list. In case of empty candidate primer lists, candidate tuple lists or tuple sets, refinements are done if possible. Finally, results are returned by mail.

The most important steps of our software can be found in the flowchart of Figure 2.

### Selection of restriction sites

To select the optimal set of restriction sites for the global mode, the genomic region of interest is first divided into equally sized intervals (one for each primer pair to be designed). Second, each interval is scanned for the presence of restriction sites for the user-defined restriction enzyme. Third, within each interval, the restriction site with the least distance to the center of the interval is chosen. It should be noted that this step is omitted in case a targeted primer design is required by the user. Instead of automatic selection of restriction sites by our algorithm, the user has to provide distinct sequences. These sequences have to contain at least one restriction site each for the target enzyme. The number of sequences provided must correspond to the number of primer tuples to be designed.

### Primer Enumeration

For the set of restriction sites from step 1, or as provided by the user, all valid upstream, downstream and, if desired, hybridization probe primers within a scan window are enumerated, considering the user-defined parameters for GC-content, melting temperature and self alignment cut-offs. The scan window is restricted by the user-defined parameters for maximum and minimum distance of a primer to the restriction site and maximum and minimum primer length. The hybridization probe has to be located between the restriction site and the scan region for the upstream primer on the other (reverse) DNA strand and these two scan regions must not overlap. The reverse primer, of course, has to be also located on the other (reverse) DNA strand in a scan window of equal size than the forward primer scan window but downstream of the restriction site (see Figure 1c).

In order to avoid redundant computations, we precompute the alignments of all primer enumeration regions with itselves and the relevant combinations of cross-region alignments. Thereafter, we can extract a sub-alignment for each primer self- and pair-alignment (used during primer tuple picking) in linear time. Primer melting temperature calculation was done using the method of SantaLucia *et al. *[10,11]. This method outperforms the frequently used Breslauer *et al. *method [12] in accuracy (data not shown) on a set of experimentally determined oligonucleotide melting temperatures [13].

As already mentioned in the introduction, the 3C methodology generates four different ligation products for interacting DNA segments (see Figure 1a). For this reason, the 3C protocol only uses the upstream primers (and the TaqMan probes, if desired) of the primer pairs to quantify interaction frequencies. The downstream primers are only used to control digestion efficiencies before and after the restriction step. This is a critical test to assure proper digestion of each site to be studied.

A primer is considered valid if: all user-defined requirements are met, the primer does neither contain repetitive elements nor restriction sites within the binding region, and no mispriming is found in the genome under study. Additionally, the maximum length of mononucleotide repeats is restricted to 4 bases. Primer misprimings are checked in the target genome. The overall idea of our extended mispriming checks is to prune primers having misprimings, which are sufficiently close to any restriction site of the target enzyme, since these misprimings (after the re-ligation step) could amplify the wrong hybrid template along with the true positive one.

In case no valid primers can be found around any restriction site, a fall-back mechanism will repeat this enumeration procedure using the 'next best restriction site' if present. In global mode, this 'next best site' is chosen from the list of restriction sites for a sequence region. This list is ordered by increasing distance to interval mean. In targeted mode, the same strategy is used. Here, each interval corresponds to one sequence provided by the user. This procedure is iterated until at least one valid primer pair has been found for a restriction site of the respective interval/sequence or no restriction sites remain to be scanned in this interval/sequence. In case of an interval/sequence where no valid primer pairs can be found (meaning that at least one of the verified candidate primer lists is empty), the program terminates with an error message.

By ordering the restriction sites by increasing distance to the interval/sequence mean, we perform a directed search from the most-centered restriction site in each interval/sequence towards the least-centered.

This search strategy makes sense in 'global mode', given the fact that we are looking for primers which are most homogeneously distributed on the genomic region of interest. In 'targeted mode', this mechanism provides a fall-back but can be omitted by providing sequences containing only one restriction site each.

### Primer Tuple Picking

In order to find the best set of primer tuples, we first have to create and screen primer tuples from the lists of valid primers for each restriction site to be studied. Each primer tuple consist of: one forward primer, one reverse primer and (if desired) a TaqMan probe (see Figure 1c). An acceptable candidate primer tuple to be used for screening has low intra-tuple alignment values to all other primers of the sample tuple (below user-defined threshold). Additionally, the difference in melting temperatures between forward and reverse primer has to be below the user-defined threshold and the difference between the melting temperature of the TaqMan probe (if desired) and the forward and reverse primers has to be above a user-defined threshold.

**Figure 3:**
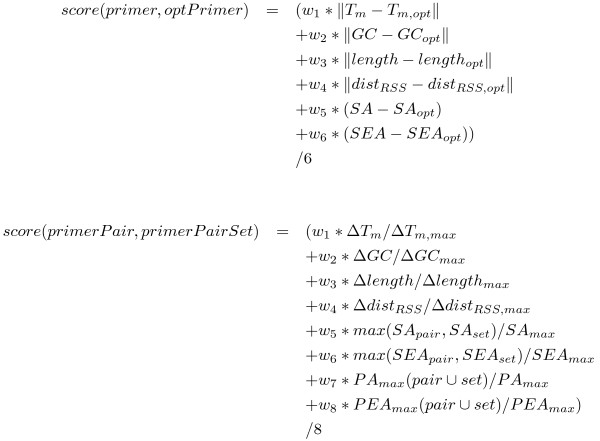
**Scoring Function**. The scoring function used to compare primers and primer tuples. Each component of the scoring function is weighted and weights are refinable by the user. The components are (in order of appearance): difference in melting temperature(s) (*T*_*m*_) to ideal melting temperature(s) (*T*_*m*, *opt*_), difference in GC content(s) (*GC*) to ideal GC content(s) (*GC*_*opt*_), difference in primer length(s) (*length*) to ideal length(s) *($length_{opt}$)*, difference in distance(s) to the restriction site(s) (*dist*_*RSS*_) to ideal distance (*dist*_*RSS*, *opt*_), difference in self alignments (*SA*) to optimal self alignment value (*SA*_*opt*_), difference in self end alignments (*SEA*) to ideal self end alignment value (*SEA*_*opt*_), and (only for primer pairs): difference in pair alignments of the candidat tuple and the set to which the candidate could be added (*PA*_*max*_(*pair *∪ *set*)/*PAmax*) and difference in pair end alignments of the candidat tuple and the set to which the candidate could be added (*PEA*_*max*_(*pair *∪ *set*)/*PEA*_*max*_), respectively.

Using these lists of valid primer tuples, one for each restriction site to be studied, we 'only' have to choose the most homogeneous set of primer tuples containing one primer tuple per interval/restriction site. This indeed is the most demanding task in our primer-design process, since the exhaustive enumeration of all primer pair sets is *O*(*m*^*n*^) with *m *the average number of valid primer tuples per restriction site in an interval and *n *the number of intervals/sequences to be studied. The worst case number of valid primer tuples per restriction site is j * k * l with *j *and *k *being the number of valid forward and reverse primers respectively and *l *being the number of valid TaqMan probes. We made our software more scalable by avoiding an exhaustive enumeration of all primer tuple sets. We incrementally build primer tuple set candidates by adding the 'most suitable' primer tuple as defined by our scoring function (Figure 3) at each step (see Figure 1d). This scoring function includes various parameters of the primer which are also frequently used by other primer design algorithms. Each parameter is weighted and weights are defined by the user - details can be found in the caption of Figure 3.

Our strategy is to sort the array of primer lists by the number of primer tuples in decreasing order. We start with the largest list of valid primer tuples and create a new primer tuple set of size one for each tuple and extend each set by the 'most suitable' primer tuple from the second largest list and so forth. The 'most suitable' primer tuple is defined as the tuple with the most similar score to the average score of the current primer tuples set, while satisfying cross-tuple alignment constraints as defined by the user. The score of a single primer tuple here corresponds to the score between this primer tuple and a virtual optimal primer tuple satisfying all design constraints best.

While creating these candidate primer tuple sets, only the one having smallest score and highest homogeneity is retained after each set enumeration. This procedure can be executed in parallel by splitting the first list of primer tuples into *x *shares of approximate equal size and running a unique thread for each share. After all threads terminate, the set with lowest score and highest homogeneity is returned as the optimal set of primer tuples. It should be noted that this threading does not influence the outcome of our algorithm but significantly speeds up the screening phase.

In case no valid primer tuples can be created for one restriction site, this sequence region is refined and the 'next closest' restriction site (if existing) with respect to the interval mean is scanned for valid primers, and pairs are created and checked for validity.

## Results and Discussion

### The web interface to 3C primer design

For convenience, we created a web interface to our 3C primer design method which can be found at http://www.pristionchus.org/3CPrimerDesign/.

The user is able to operate our primer design software in two different modes: targeted search and global search. In a targeted search, the program will design the best set of primer pairs for a set of sequence regions which can be provided as multi fasta input (one fasta sequence per sequence region). Each sequence region must contain one or more restriction sites, to screen for suitable pairs. This screen is done, as previously described, starting with the most centered restriction site of each region. In a global search, only one sequence has to be provided. This sequence is evenly split into a number of sequence regions (one for each primer tuple). Again, each sequence region is screened for valid pairs by starting with the most centered restriction site.

The list of program and score function parameters is divided into three sections: basic parameters, advanced parameters and weightings. The basic parameters section comprises: the job id, the sequence on which to design primers, the numbers of primers to design, the desired restriction enzyme, the organisms for which to design primers (this is required for primer mispriming scans) and the mail address to which the program output will be sent.

As a minimal requirement, the user has to completely specify the parameters in the basic parameters section. Currently, the organism list encompasses *Caenorhabditis elegans*, *Pristionchus pacificus*, *Drosophila melanogaster*, *Mus musculus*, *Homo sapiens *and *Saccharomyces cerivisiae*. This list can be extended upon request. The advanced parameters section comprises various parameters relevant for primer design in general and 3C primer design in particular. Weights for score calculations can be adjusted in the corresponding section. The user submits the 3C primer design job after specifying all relevant parameters. The program output is delivered as an e-mail, which reports the best primer set.

A detailed description of the web interface along with examples can be found at the website.

### Runtime benchmark

We performed a runtime benchmark to estimate the number of primer tuples, which could be computed in a reasonable time frame. This was done for the *S. cerevisiae *chromosome I using 'global mode'. For this benchmark, we used the default parameters of our web interface. The number of primer tuples to return were set to: 2, 4, 8 and 16 respectively. We did this benchmark for both: primer pairs (excluding TaqMan probes) and primer triples. All benchmarks were performed on a standard workstation: MacPro dual-quadcore 2.26 GHz having 16 GB of RAM. All runtimes reported are realtime in milliseconds as determined by the GNU time tool.

**Figure 4:**
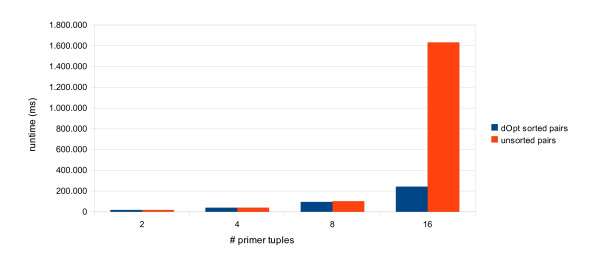
**Runtime Benchmark for Primer Tuples not containing TaqMan probes**. The runtime (realtime) is shown in milliseconds for the computation of 2, 4, 8 and 16 optimal primer tuples not including TaqMan probes. Another benchmark was done using an optional sorting of the lists of primer tuples prior to screening. In this case, screening is done using a binary search and terminating at the first set of tuples satisfying all constraints (per working thread).

As can be seen from Figure 4, our method is quite fast in creating a set of primer pairs (excluding TaqMan probes). An optional sorting of the list of primer tuples combined with a binary search in these sorted lists would drastically improve the runtime at 16 tuples and above. We did not test any larger problem sizes since computation times of > 30 minutes are untenable for a web service. Our web service implements the more comprehensive search strategy (orange bars).

**Figure 5:**
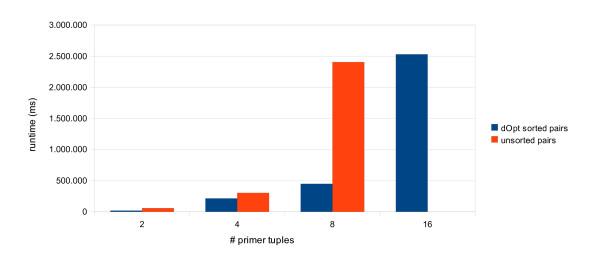
**Runtime Benchmark for Primer Tuples containing TaqMan probes**. The runtime (realtime) is shown in milliseconds for the computation of 2, 4, 8 and 16 optimal primer tuples including TaqMan probers. Another benchmark was done using an optional sorting of the lists of primer tuples prior to screening. In this case, screening is done using a binary search and terminating at the first set of tuples satisfying all constraints (per working thread).

A similar trend is observable for primer triples (Figure 5). However, the runtime increase for the standard search strategy occurs one step earlier (8 triples). The unsorted computation of 16 tuples took more than 12 hours (not shown).

### Experimental procedures

**Figure 6:**
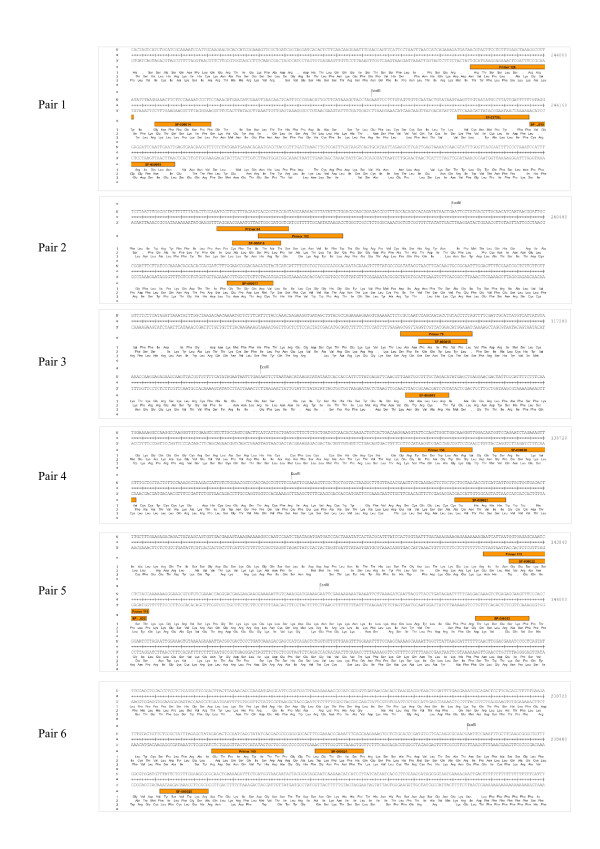
**Genomic target loci on yeast chromosome III**. The six genomic target loci on chromosome III, which were selected for experimental primer validation, are shown. Primer positions are shown as orange boxes. forward primers are left to the EcoRI restriction site. Forward primers were used for primer pairing experiments.

We performed a comparative primer design experiment based on a previously published primer set for chromosome III in *Saccharomyces cerevisiae *[14]. For this evaluation, we randomly picked six target regions: three from the GC-rich domain and three from the AT-rich domain of yeast chromosome III (see Dekker [14] for details). For each target site, we extracted 110 bp up- and downstream of the EcoRI restriction site. We used our 3PD program in 'Targeted Mode' with default parameters. No TaqMan probes were selected and 'Target Organism' was set to '*S. cerevisiae*'. The primers by Dekker [14] (identifiers for AT-rich region: 79,136 and 119; identifiers for GC-rich region: 84, 126, 145) and all 3PD primers were synthesized by BioTeZ, Berlin-Germany. The genomic locations of all primers are shown in Figure 6. We compared all pairwise combinations of our upstream primers to the ones from Dekker *et al. *The experiment was performed on a 'control template' from yeast genomic DNA. 600 *μ*g of genomic yeast DNA were extracted and purified with the 'QIAGEN Genomic-tip 500/G' kit. This gDNA was digested with EcoRI and randomly religated with T4 DNA ligase. Quantitative PCRs with SYBR Green I dye were set up as a two-fold dilution series from 500 ng to ≈ 30 ng in five dilution steps. The standard curves for each non-redundant primer pairing of our primer set as well as for the respective primer set of Dekker are shown in Additional File 1. In short, we did not observe any significant differences between the two primer sets.

## Conclusions

In this work, we have developed the first fully integrated primer design suite for chromatin conformation capture (3C) experiments. Our method is able to create primer setups for all three 3C variants. We have discussed the critical steps and constraints in 3C primer design and how our program implements them. Finally, we have introduced a web interface to our 3PD software and we have provided a runtime benchmark on real-world problem sizes. We demonstrated that our primers perform as good as hand-crafted primers on a control template from random ligations of yeast genomic DNA. Our software is freely available to academic users as a web server at: http://www.pristionchus.org/3CPrimerDesign/.

## Availability and requirements

• *Project name*: 3C Primer Design

• *Project home page*: http://www.pristionchus.org/3CPrimerDesign/

• *Operating system(s)*: Platform independent

• *Programming language*: Java

• *Other requirements*: Any web browser supporting forms

• *License*: The web interface is freely available to academic users

• *Any restrictions to use by non-academics*: Licence needed

## Authors' contributions

CD conceived and outlined the 3C primer design approach. SF developed all software and carried out all experiments. CD and SF wrote and approve the manuscript.

## Supplementary Material

Additional file 1 **qPCR standard curves**. The left column shows the 3PD primer pairings and the right column shows the Dekker primer pairings. Primer number are the same as in Figure 6. Five dilution steps are shown in duplicate. Click here for file
