# Supplementary figures and images for: 3PD: Rapid design of optimal primers for chromosome conformation capture assays
Source: BMC Genomics. 2009 Dec 29;10:635. doi: 10.1186/1471-2164-10-635 (PMC2811132; doi:10.1186/1471-2164-10-635)

3PD Primers

Dekker Primers

14/16

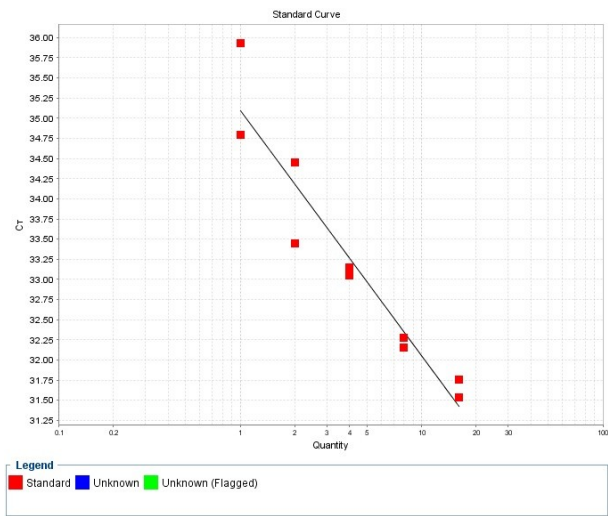

126/84

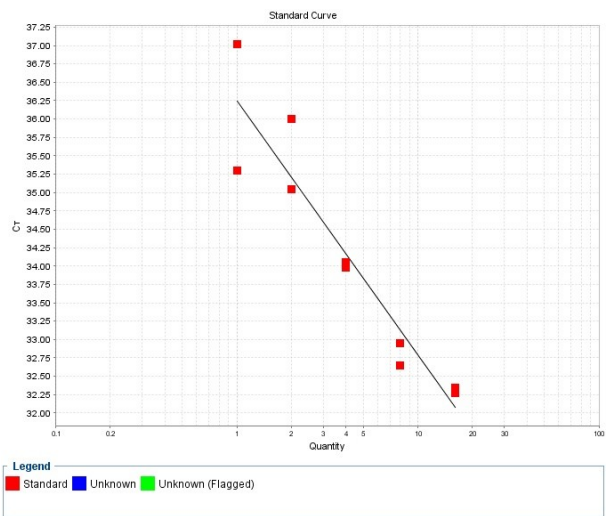

14/18

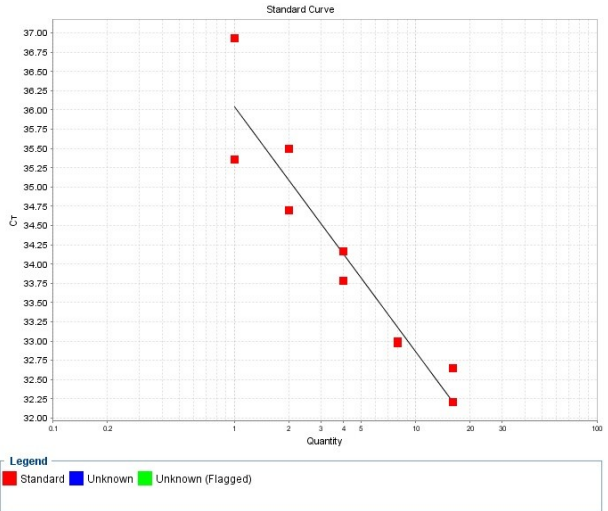

126/79

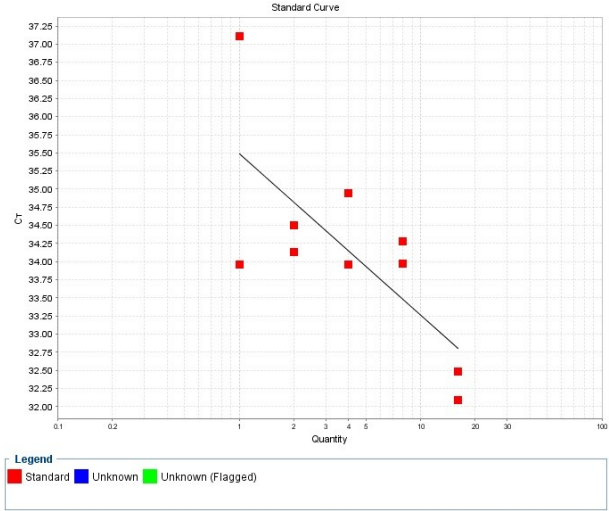

14/20

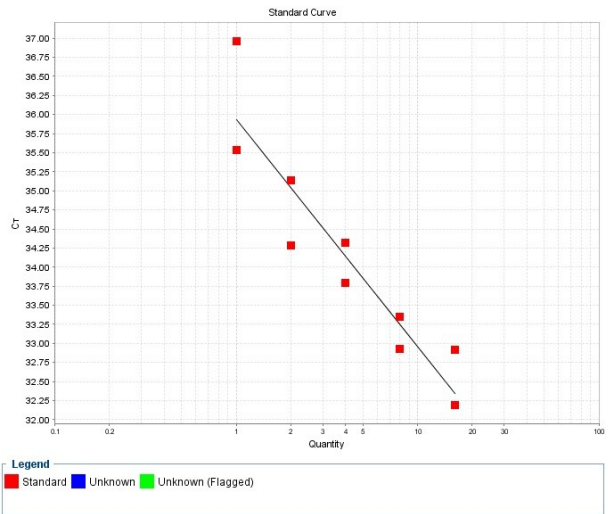

126/136

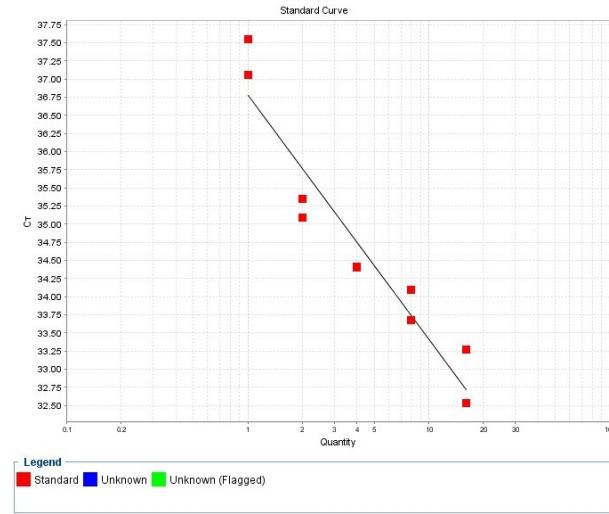

3PD Primers

Dekker Primers

14/22

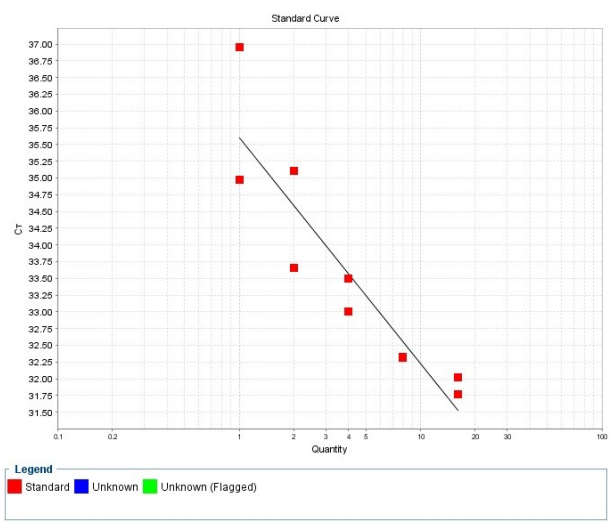

126/119

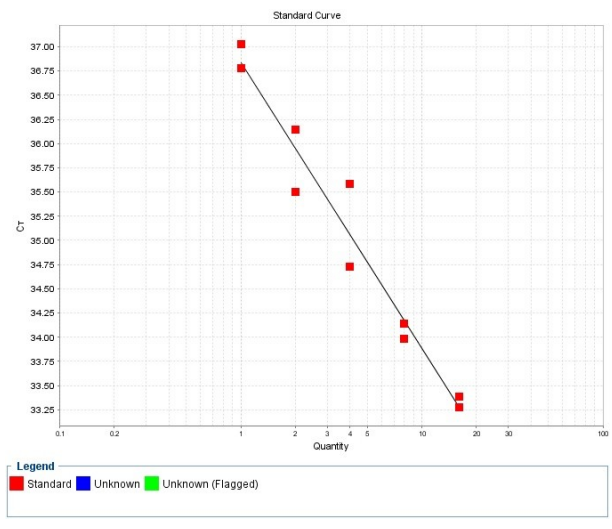

14/24

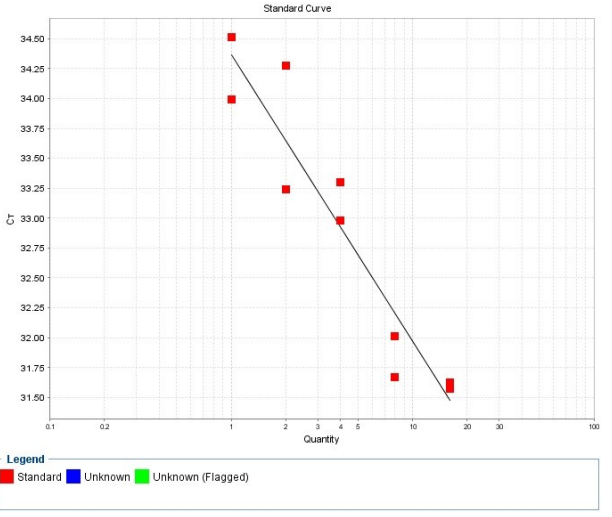

126/145

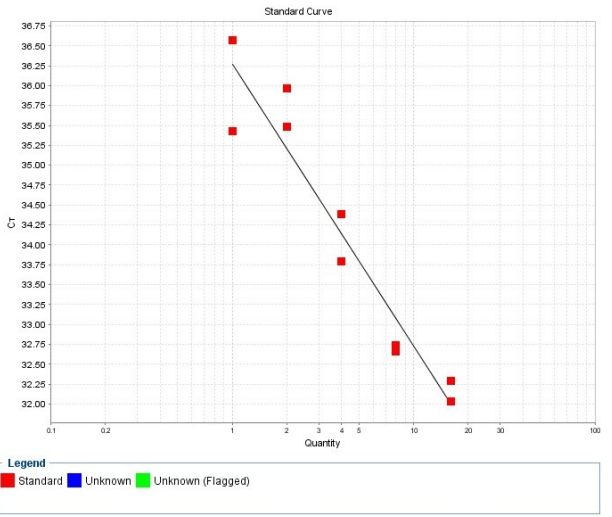

16/18

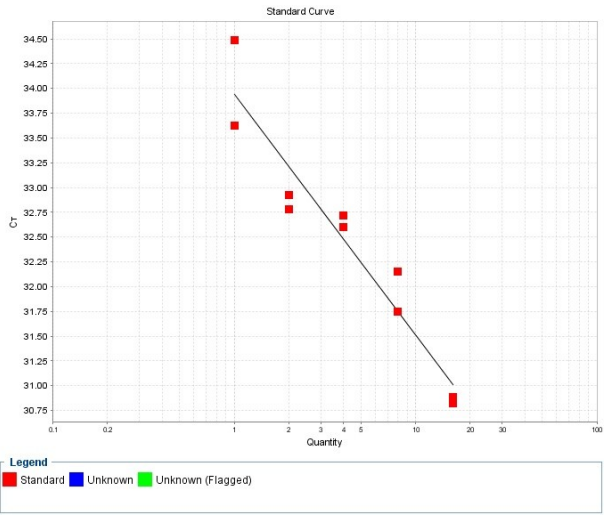

84/79

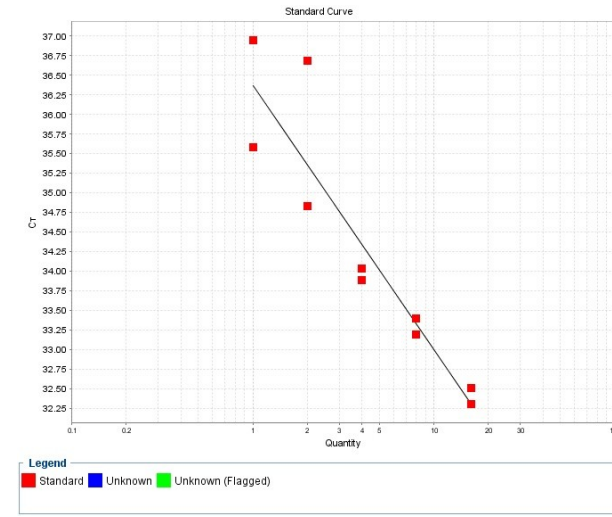

16/20

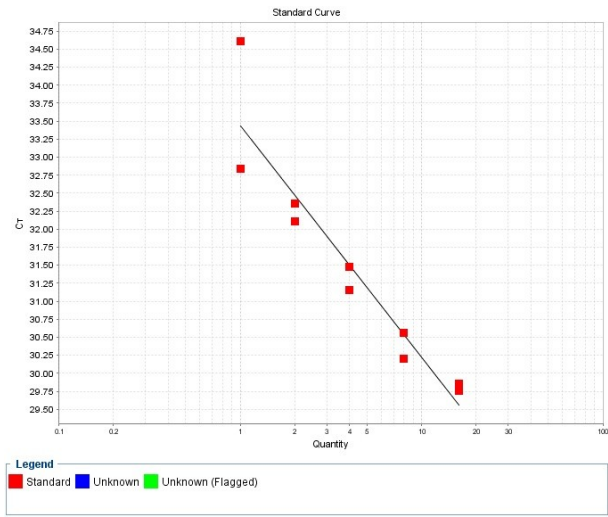

84/136

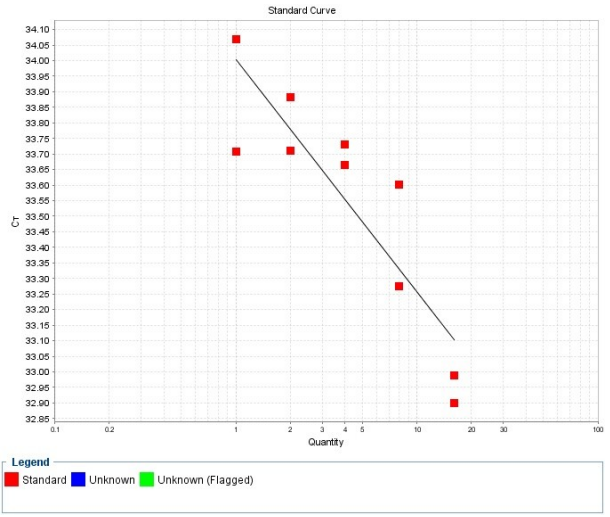

16/22

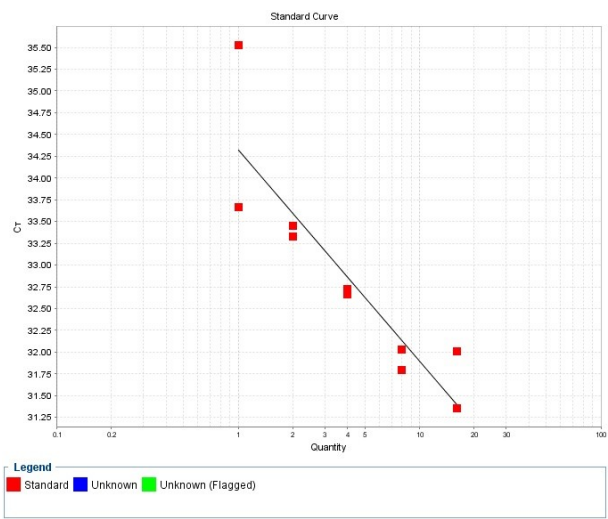

84/119

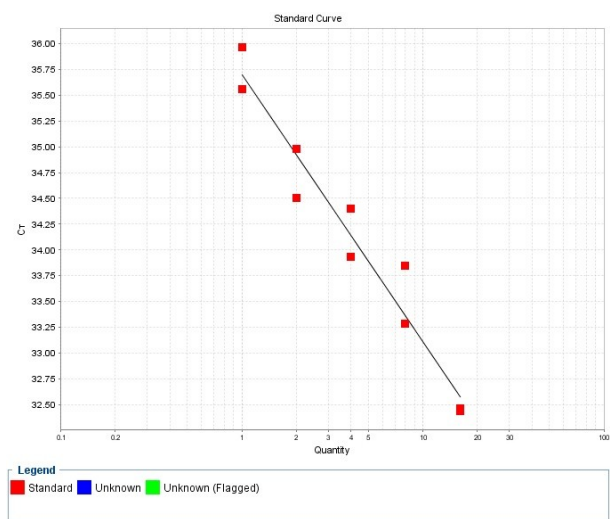

16/24

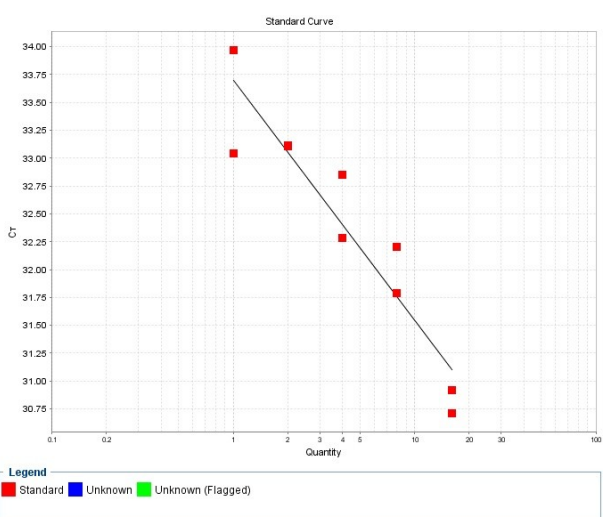

84/145

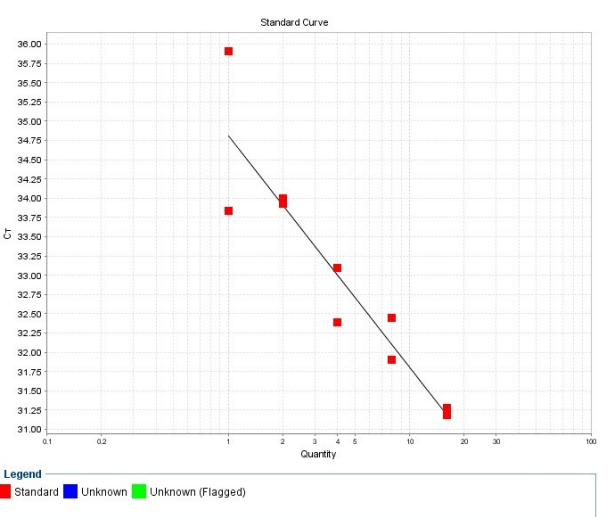

18/20

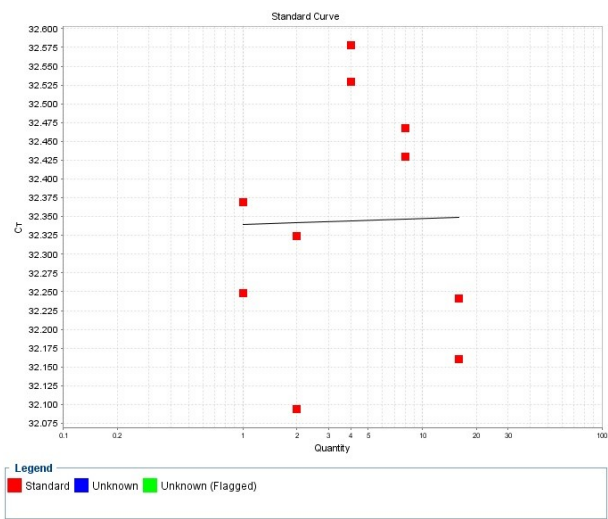

79/136

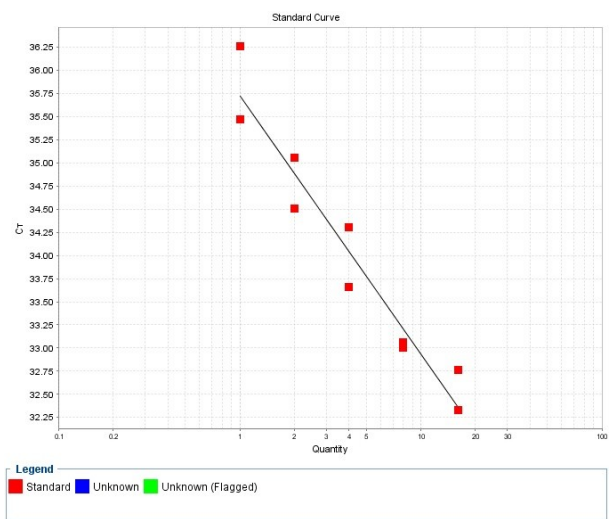

18/22

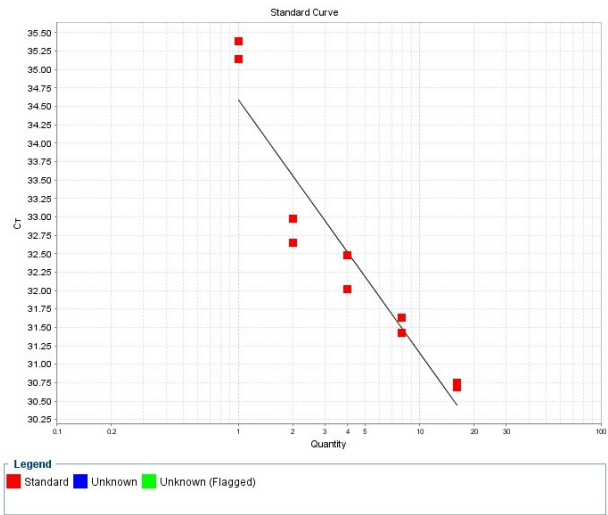

79/119

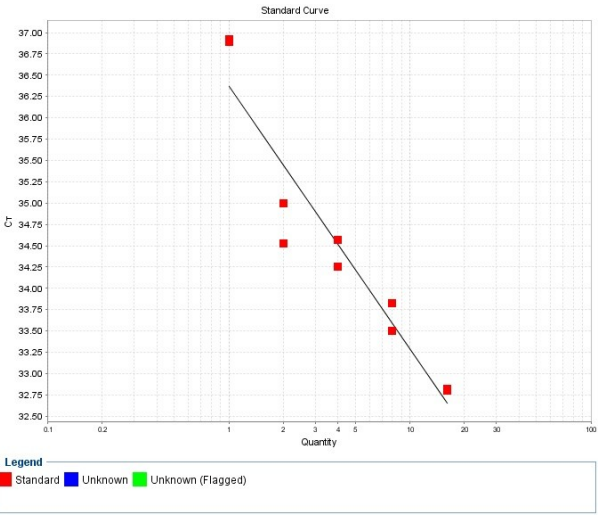

18/24

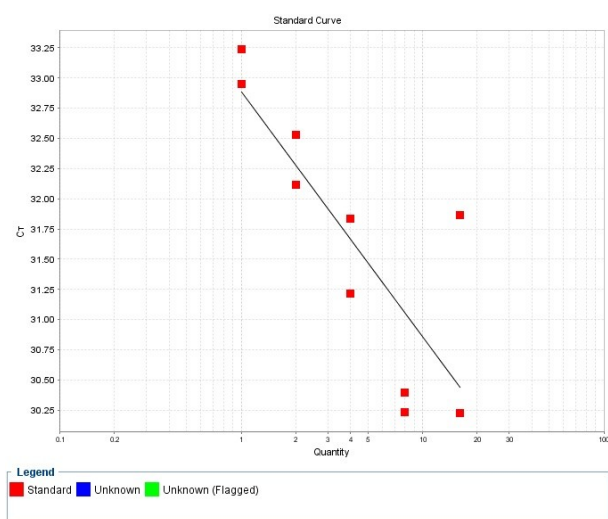

79/145

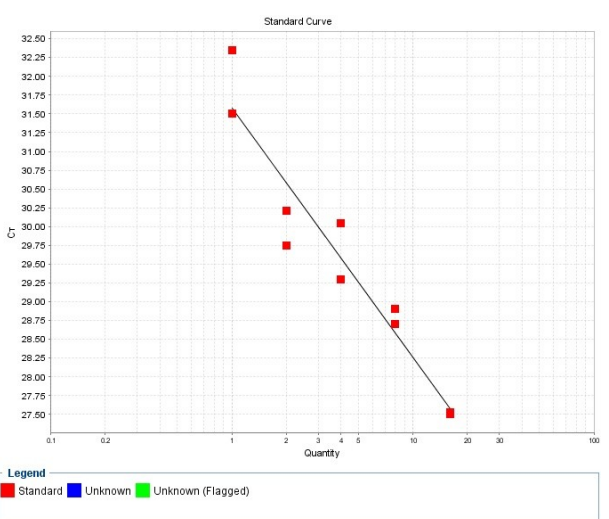

3PD Primers

Dekker Primers

20/22

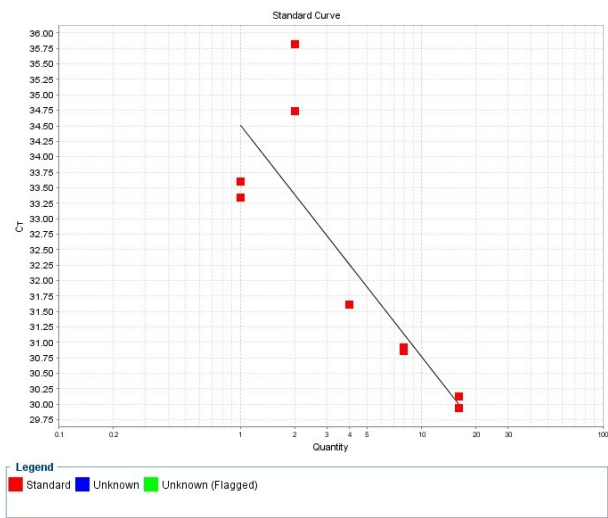

136/119

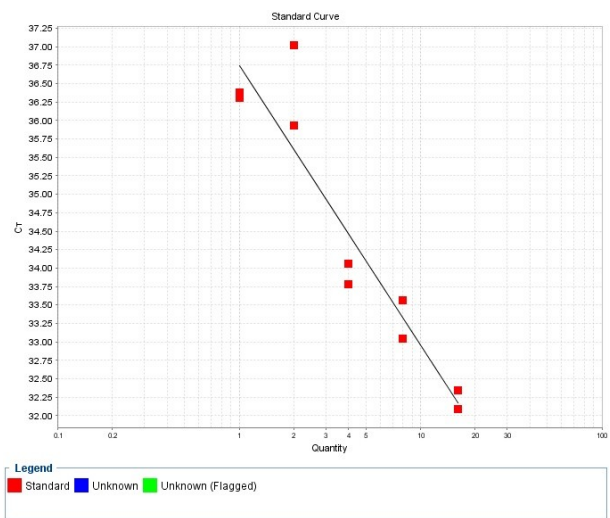

20/24

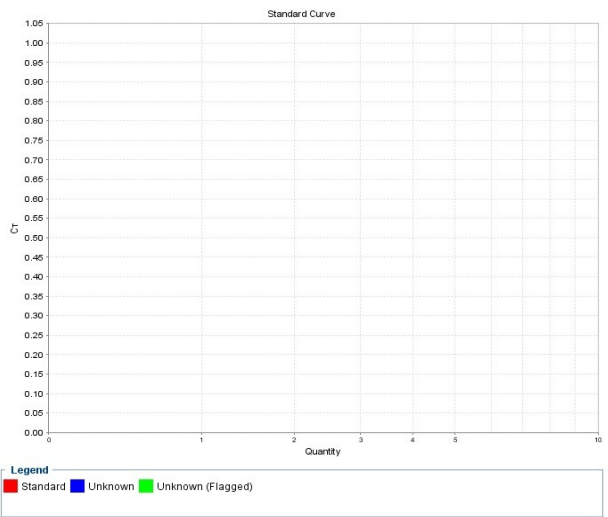

136/145

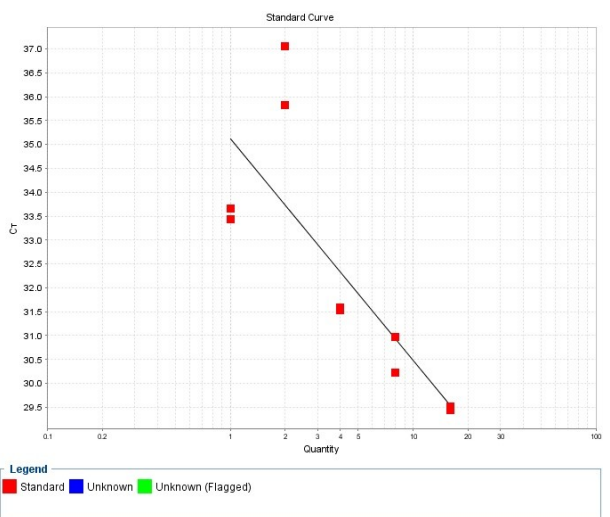

22/24

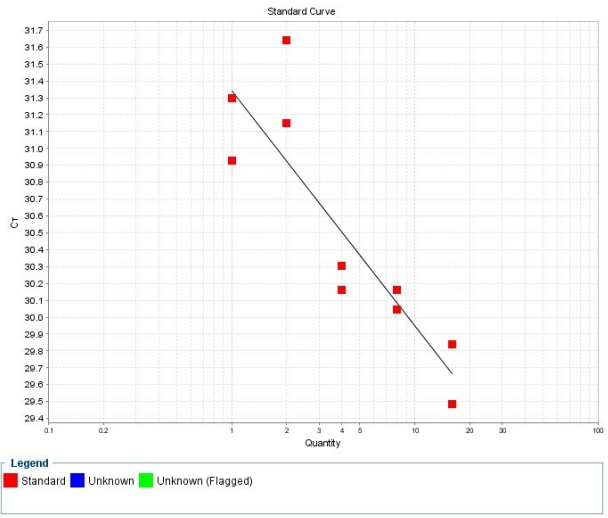

136/145

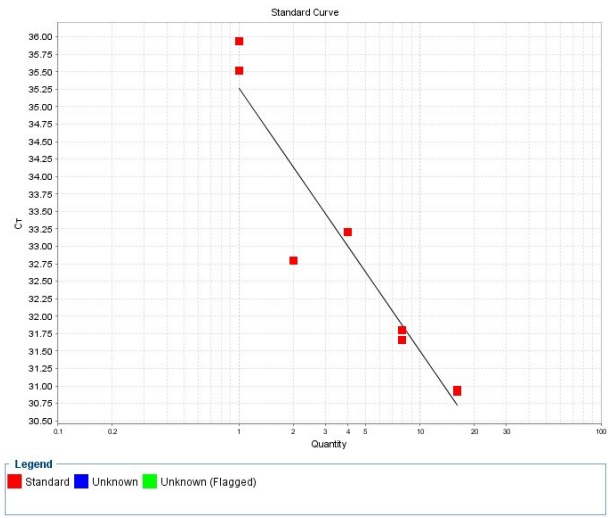

Supplement: Additional file 1 — qPCR standard curves. The left column shows the 3PD primer pairings and the right column shows the Dekker primer pairings. Primer number are the same as in Figure 6. Five dilution steps are shown in duplicate. [file 1471-2164-10-635-S1.PDF]
